# A Novel Toxicokinetic Modeling of Cypermethrin and Permethrin and Their Metabolites in Humans for Dose Reconstruction from Biomarker Data

**DOI:** 10.1371/journal.pone.0088517

**Published:** 2014-02-26

**Authors:** Jonathan Côté, Yvette Bonvalot, Gaétan Carrier, Caroline Lapointe, Uwe Fuhr, Dorota Tomalik-Scharte, Bertil Wachall, Michèle Bouchard

**Affiliations:** 1 Département de santé environnementale et santé au travail, Chaire d'analyse et de gestion des risques toxicologiques and Institut de recherche en santé publique de l'Université de Montréal (IRSPUM), Université de Montréal, Montreal, Quebec, Canada; 2 Environmental Health Program, Health Canada, Longueuil, Quebec, Canada; 3 Department of Pharmacology, University of Cologne, Clinical Pharmacology Unit, Köln, Germany; 4 Infectopharm Arzneimittel und Consilium GmbH, Heppenheim, Germany; Memorial Sloan Kettering Cancer Center, United States of America

## Abstract

To assess exposure to pyrethroids in the general population, one of most widely used method nowadays consists of measuring urinary metabolites. Unfortunately, interpretation of data is limited by the unspecified relation between dose and levels in biological tissues and excreta. The objective of this study was to develop a common multi-compartment toxicokinetic model to predict the time courses of two mainly used pyrethroid pesticides, permethrin and cypermethrin, and their metabolites (*cis*-DCCA, *trans*-DCCA and 3-PBA) in the human body and in accessible biological matrices following different exposure scenarios. Toxicokinetics was described mathematically by systems of differential equations to yield the time courses of these pyrethroids and their metabolites in the different compartments. Unknown transfer rate values between compartments were determined from best fits to available human data on the urinary excretion time courses of metabolites following an oral and dermal exposure to cypermethrin in volunteers. Since values for these coefficients have not yet been determined, a mathematical routine was programmed in MathCad to establish the possible range of values on the basis of physiological and mathematical considerations. The best combination of parameter values was then selected using a statistic measure (reliability factor) along with a statistically acceptable range of values for each parameter. With this approach, simulations provided a close approximation to published time course data. This model allows to predict urinary time courses of *trans*-DCCA, *cis*-DCCA and 3-PBA, whatever the exposure route. It can also serve to reconstruct absorbed doses of permethrin or cypermethrin in the population using measured biomarker data.

## Introduction

Pyrethroids are synthetic insecticides increasingly used in agriculture, which tend to replace organophosphate and carbamate insecticides. Pyrethroids are considered to be neurotoxic, according to *in vitro* studies and data in workers [1], [2], [3]. In *in vitro* studies, some pyrethroids (namely permethrin, cypermethrin and deltamethrin) were also shown to cause genotoxic alterations (DNA addcuts or damage) [4], [5], [6], [7], [8]. Certain pyrethroids (cyfluthrin, permethrin and cypermethrin) were further documented to exhibit endocrine disruption properties, such as anti-androgenic effects [8], [9].

In Canada and elsewhere, pyrethroids are used in many cultures, thus with a potential to contaminate fruits, vegetables and cereals. Dairy products may also contain residues of these insecticides, because of their usage for the treatment of animals and farms [10]. Residues of pyrethroids have further been found in animal fat and even olive oil [11], [12]. Given their extensive application in agriculture and farming, contaminated food appears as the primary source of exposure in urban populations [13], [14]. However, pyrethroids are also used in some workplaces and in homes to control for insect pests (ants, cockroaches, mosquitos, etc…); there is thus a potential for exposure by other routes in the general population, such as the dermal route [15], [16], [17].

Because of chronic exposure to these pesticides through the diet in the general population and the possibility of dermal exposure following certain home treatments, scientists and government agencies have recognized the importance of documenting exposure to these products. For this type of product, biological monitoring is recognized as the most appropriate method for assessing exposure. In this context, extensive data on concentrations of urinary metabolites of pyrethroids were collected as part of the Canadian Health Measures Survey (CHMS) and the NHANES surveys [18], [19] and other Quebec studies on human biomonitoring [20], [21]. According to the latter recently published biomonitoring data in Quebec adults and children, permethrin and cypermethrin are major contributors of the overall exposure to pyrethroids in the general environment [20], [21].

Although biomonitoring is now largely used to assess exposure to this type of pesticides, interpretation of biomomintoring data is currently limited by the undefined relationship between dose or concentration in the environment and levels in biological tissues or excreta. An approach that can help the interpretation of biological data is to develop toxicokinetic models for the pyrethroids of interest and their exposure biomarkers in the body as done for other pesticides [22], [23], [24], [25], [26]. These toxicokinetic models can then be used to reconstruct the daily rates of absorption corresponding to the observed biomonitoring data [27].

Mirfazaelian et al. [28] proposed a physiologically-based pharmacokinetic (PBPK) model for a pyrethroid, deltamethrin, but it is a rat model that does not describe the kinetics of urinary metabolites of this pyrethroid. Tornero-Velez et al. [29] have subsequently modified this rat PBPK model to predict dosimetry during maturation, although it cannot be used as such to estimate the daily exposure or absorbed doses in humans from biomarker data. Tornero-Velez et al. [30] recently published a human PBPK model to describe the kinetics of permethrin, based on a rat model, and linked this model to a one-compartment model describing the *trans*- and *cis*-DCCA metabolites.

Using a different modeling approach, we developed at the same time a human data-based toxicokinetic model of both permethin and cypermethrin that relates absorbed doses to common biomarkers of exposure, *cis*- and *trans*-2,2-(dichlorovinyl)-2,2-dimethylcyclopropane carboxylic acids (*cis*- and *trans*-DCCA) and 3-phenoxybenzoic acid (3-PBA), as a function of the exposure route and temporal scenarios. The first phase of the study was to establish a common conceptual model of the kinetics of both permethrin and cypermethrin based on available human time-course data, use a new routine to test the various possibilities of parameter values, and verify the goodness of fit of the model to experimental kinetic data in volunteers exposed to either of those pyrethroids under certain controlled conditions.

## Materials and Methods

### Data used for model development and evaluation

The key study used for model development is the study of Woollen et al. [31] on the detailed individual time courses of cypermethrin metabolites, *cis*- and *trans*-DCCA, 3-PBA, and 4-hydroxy-3-phenoxybenzoic acid (4-OH-3-PBA), in the urine of volunteers over a 120-h period following a single oral administration of either 3.3 mg of cypermethrin (ratio *cis*∶*trans* of 50∶50; dissolved in ethanol and administered on a sugar cube) (n = 6) or dermal application of 31 mg on 800 cm^2^ of skin (*cis*∶*trans* ratio of 56∶44; mixed with surfactants and wetting agents, and diluted in soya bean oil; application area cleaned 8 h post-application) (n = 6; 4 subjects being the same as for the oral administration). Metabolism and time course data in animals exposed to permethrin and cypermethrin also served to corroborate modeling assumptions [32], [33], [34], [35], along with the time course of permethrin in the serum of a patient intoxicated with permethrin [36].

For model evaluation, the different sets of data of Tomalik-Scharte et al. [16] on the individual time courses of DCCA metabolite in the urine of volunteers following different dermal application scenarios of permethrin were used. These include kinetic data in healthy volunteers following application on the scalp of an ethanolic solution containing 0.5% permethrin or a whole body application of a cream containing 5% permethrin as well as results in scabies patients following a whole body application of a cream containing 5% permethrin (n = 6 per group). The quality of these data kindly provided by the authors allowed evaluating model simulations of the kinetics of permethrin and cypermethrin and their metabolites.

### Conceptual and functional representation of the model

**Figure 1:**
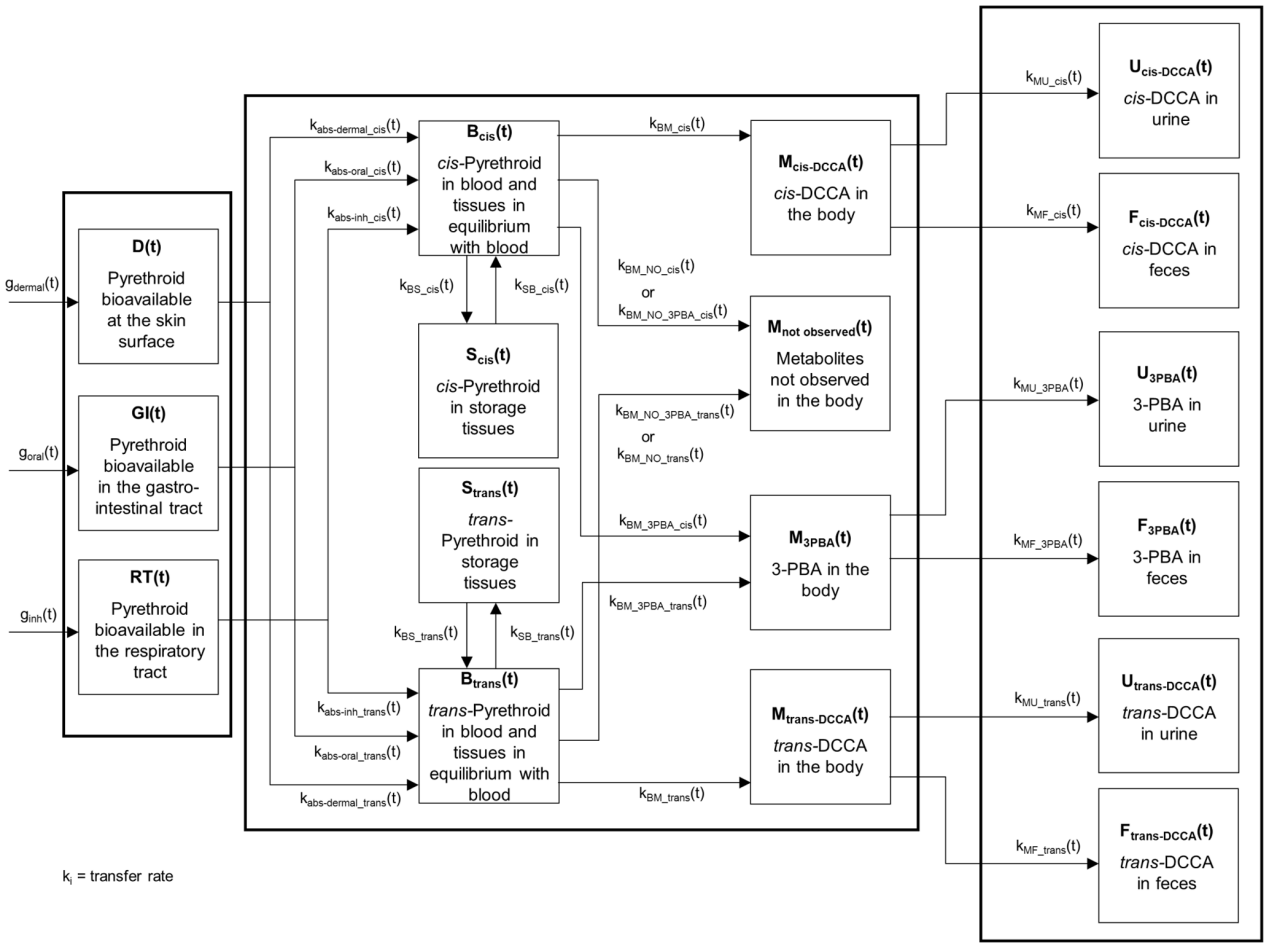
Model conceptual representation. Model conceptual representation of the kinetics of *cis*- and *trans*-permethrin and cypermethrin and their *trans*-DCCA, *cis*-DCCA and 3-PBA metabolites. Symbols are described in Table 1.

The kinetics of permethrin and cypermethrin and their metabolites was modeled using a same multi-compartment dynamical system because of their common metabolism (production of common breakdown metabolites) and similar observed time course of metabolites. The conceptual model is depicted in Figure 1 and symbols and abbreviations are described in Table 1. Compartments represent the burdens or cumulative excretion of these pyrethroids or their metabolites as a function of time and arrows stand for the rates of transfer or biotransformation of these pyrethroids and their metabolites. The evolution of burdens is described mathematically by differential equations where the rate of change in the amounts in each compartment (dX_i_(t)/dt) (on a molar basis) is the difference between the incoming and outgoing transfer rates (see Appendix S1 for differential equations). Since there are two isomeric forms of the parent compound (*cis*- and *trans*-), the evolution of burdens of each isomeric form is represented separately, along with the *cis*- and *trans*-DCCA metabolites resulting from the breakdown of the parent compound. The 3-PBA metabolite counterpart is modeled to originate from the addition of both the *cis*- and *trans*- isomers of permethrin and cypermethrin. The model was built while ensuring conservation of mass (in moles), hence at all times, the dose was equal to the sum of burdens in the different compartments (parent compound and metabolites) as well as those accumulated in excreta since exposure.

**Table 1:** Description of symbols used in the conceptual and functional representation of the kinetic model of permethrin and cypermethrin and their metabolites.

Parameters	Definitions
g_oral_(t)	Oral dose (mol) bioavailable per unit of time which can describe time varying inputs
g_dermal_(t)	Dermal dose (mol) bioavailable per unit of time which can describe time varying inputs
g_inh_(t)	Inhalation dose (mol) bioavailable per unit of time which can describe time varying inputs
D(t)	Amounts of *cis*- and *trans*-permethrin or cypermethrin (mol) bioavailable at skin surface as a function of time
GI(t)	Amounts of *cis*- and *trans*-permethrin or cypermethrin (mol) bioavailable in the gastrointestinal tract as a function of time
R(t)	Amounts of *cis*- and *trans*-permethrin or cypermethrin (mol) bioavailable in the respiratory tract as a function of time
B_cis_(t)	Burden of *cis*-permethrin or cypermethrin (mol) in blood and tissues in dynamical equilibrium with blood as a function of time
B_trans_(t)	Burden of *trans*-permethrin or cypermethrin (mol) in blood and tissues in dynamical equilibrium with blood as a function of time
S_cis_(t)	Burden of *cis*-permethrin or cypermethrin (mol) in storage tissues (mol) as a function of time
S_trans_(t)	Burden of *trans*-permethrin or cypermethrin (mol) in storage tissues (mol) as a function of time
M_cis-DCCA_(t)	Body burden of *cis*-DCCA (mol) as a function of time
M_trans-DCCA_(t)	Body burden of *trans*-DCCA (mol) as a function of time
M_3PBA_(t)	Body burden of 3-PBA (mol) as a function of time
M_not observed_(t)	Body burden of non-monitored metabolites (mol) as a function of time
U_cis-DCCA_(t)	Cumulative amounts of *cis*-DCCA in urine (mol) as a function of time
U_trans-DCCA_(t)	Cumulative amounts of *trans*-DCCA in urine (mol) as a function of time
U_3PBA_(t)	Cumulative amounts of 3-PBA in urine (mol) as a function of time
QU_cis-DCCA_(t)	Urinary excretion rate of *cis*-DCCA in urine (mol) as a function of time = M_cis-DCCA_(t)×k_MU_cis_
QU_trans-DCCA_(t)	Urinary excretion rate of *trans*-DCCA in urine (mol) as a function of time = M_trans-DCCA_(t)×k_MU_trans_
QU_3PBA_(t)	Urinary excretion rate of 3-PBA in urine (mol) as a function of time = M_3PBA_(t)×k_MU_3PBA_
F_cis-DCCA_(t)	Cumulative amounts of *cis*-DCCA in feces (mol) as a function of time
F_trans-DCCA_(t)	Cumulative amounts of *trans*-DCCA in feces (mol) as a function of time
F_3PBA_(t)	Cumulative amounts of 3-PBA in feces (mol) as a function of time
f_abs_oral_cis_	Oral absorption fraction of *cis*-permethrin or cypermethrin
f_abs_oral_trans_	Oral absorption fraction of *trans*-permethrin or cypermethrin
f_abs_dermal_cis_	Dermal absorption fraction of *cis*-permethrin or cypermethrin
f_abs_dermal_trans_	Dermal absorption fraction of *trans*-permethrin or cypermethrin
k_abs_oral_cis_	Oral absorption rate of *cis*-permethrin or cypermethrin (h^−1^)
k_abs_oral_trans_	Oral absorption rate of *trans*-permethrin or cypermethrin (h^−1^)
k_abs_dermal_cis_	Dermal absorption rate of *cis*-permethrin or cypermethrin (h^−1^)
k_abs_dermal_trans_	Dermal absorption rate of *trans*-permethrin or cypermethrin (h^−1^)
k_abs_inh_cis_	Respiratory absorption rate of *cis*-permethrin or cypermethrin (h^−1^)
k_abs_inh_trans_	Respiratory absorption rate of *trans*-permethrin or cypermethrin (h^−1^)
k_BS_cis_	Blood to storage tissues transfer rate of *cis*-permethrin (h^−1^)
k_BS_trans_	Blood to storage tissues transfer rate of *trans*-permethrin (h^−1^)
k_SB_cis_	Storage tissues to blood transfer rate of *cis*-permethrin (h^−1^)
k_SB_trans_	Storage tissues to blood transfer rate of *trans*-permethrin (h^−1^)
k_BM_cis_	Biotransformation rate of *cis*-permethrin or cypermethrin into *cis*-DCCA (h^−1^)
k_BM_trans_	Biotransformation rate of *trans*-permethrin or cypermethrin into *trans*-DCCA (h^−1^)
k_BM_3PBA_cis_	Biotransformation rate of *cis*- permethrin or cypermethrin into 3-PBA (h^−1^)
k_BM_3PBA_trans_	Biotransformation rate of *trans*- permethrin or cypermethrin into 3-PBA (h^−1^)
k_BM_NO_cis_	Biotransformation rate of *cis*-permethrin or cypermethrin into non-observed *cis*-derivative metabolites (h^−1^)
k_BM_NO_trans_	Biotransformation rate of *trans*-permethrin or cypermethrin into non-observed *trans*-derivative metabolites (h^−1^)
k_BM_NO_3PBA_cis_	
	Biotransformation rate of *cis*-permethrin or cypermethrin into non-observed *cis*-phenoxy derivatives (h^−1^)
k_BM_NO_3PBA_trans_	Biotransformation rate of *trans*-permethrin or cypermethrin into non-observed *trans*-phenoxy derivatives (h^−1^)
k_MU_cis_	Transfer rate of *cis*-DCCA from the body to urine (h^−1^)
k_MU_trans_	Transfer rate of *trans*-DCCA from the body to urine (h^−1^)
k_MU_3PBA_	Transfer rate of 3-PBA from the body to urine (h^−1^)
k_MF_cis_	Transfer rate of *cis*-DCCA from the body to feces (h^−1^)
k_MF_trans_	Transfer rate of *trans*-DCCA from the body to feces (h^−1^)
k_MF_3PBA_	Transfer rate of 3-PBA from the body to feces (h^−1^)
k_metabolism_cis_	Biotransformation rate of *cis*-permethrin or cypermethrin into *cis*-metabolite forms or 3-PBA ( = k_BM_cis_+k_BM_NO_cis_) (h^−1^)
k_metabolism_trans_	
	Biotransformation rate of *trans*-permethrin or cypermethrin into *trans*-metabolite forms or 3-PBA ( = k_BM_trans_+k_BM_NO_trans_) (h^−1^)
k_elim___cis_	Total excretion rate of *cis*-DCCA = (k_MU_cis_+k_MF_cis_) (h^−1^)
k_elim___trans_	Total excretion rate of *trans*-DCCA = (k_MU_trans_+k_MF_trans_) (h^−1^)
k_elim___3PBA_	Total excretion rate of 3-PBA = (k_MU_3PBA_+k_MF_3PBA_) (h^−1^)
ω_cis_	k_BM_cis_×k_MU_cis_
ω_trans_	k_BM_trans_×k_MU_trans_
ω_3PBA_	k_BM_3PBA_×k_MU_3PBA_

The model uses specific input compartments, D(t), GI(t), RT(t), to describe the amounts of permethrin or cypermethrin bioavailable at the skin surface, the gastrointestinal tract and the respiratory tract, respectively. Tissue burdens of permethrin and cypermethrin that rapidly reach and maintain a fixed ratio with the blood burden were grouped with the blood burden in a single lumped compartment, B_cis_(t) or B_trans_(t), since all these amounts evolve in parallel. Another compartment, S_cis_(t) or S_trans_(t), regroups storage tissue burdens of permethrin and cypermethrin that are slowly returned to blood. These S(t) compartment were introduced in the model to account for the biphasic elimination kinetics of cypermethrin metabolites in the urine of volunteers orally exposed to cypermethrin [31]. The terminal elimination phase of this urinary time course curve was assumed to be due to the slow release to blood of cypermethrin accumulated in lipids or covalently bound to tissue proteins.

Compartments M_cis-DCCA_(t), M_trans-DCCA_(t) and M_3-PBA_(t) represent the body burden of the main metabolites *cis*-DCCA, *trans*-DCCA and 3-PBA while compartment M_not observed_(t) corresponds to unmeasured metabolites. This is to account for the fact that other metabolites than those used as exposure biomarkers may be formed, according to Kaneko et al. [35]; thus, one mol of 50∶50 (*cis*∶*trans*) permethrin or cypermethrin may not generate ½ mol of *cis*-DCCA, ½ mol of *trans*-DCCA and 1 mol of 3-PBA+4-OH-3-PBA.

The compartments U_cis-DCCA_(t), U_trans-DCCA_(t) and U_3-PBA_(t) represent the cumulative excretion of total *cis*-DCCA, *trans*-DCCA or 3-PBA observed in urine and F_cis-DCCA_(t), F_trans-DCCA_(t) and F_3-PBA_(t) the cumulative excretion of total *cis*-DCCA, *trans*-DCCA or 3-PBA excreted via feces.

Resolution of the differential equations simulating the kinetics of the parent compounds and their metabolites in the body generated the mathematical functions (X_i_(t)) describing the time profile of these molecules in the different model compartments.

### Determination of model parameters

**Figure 2:**
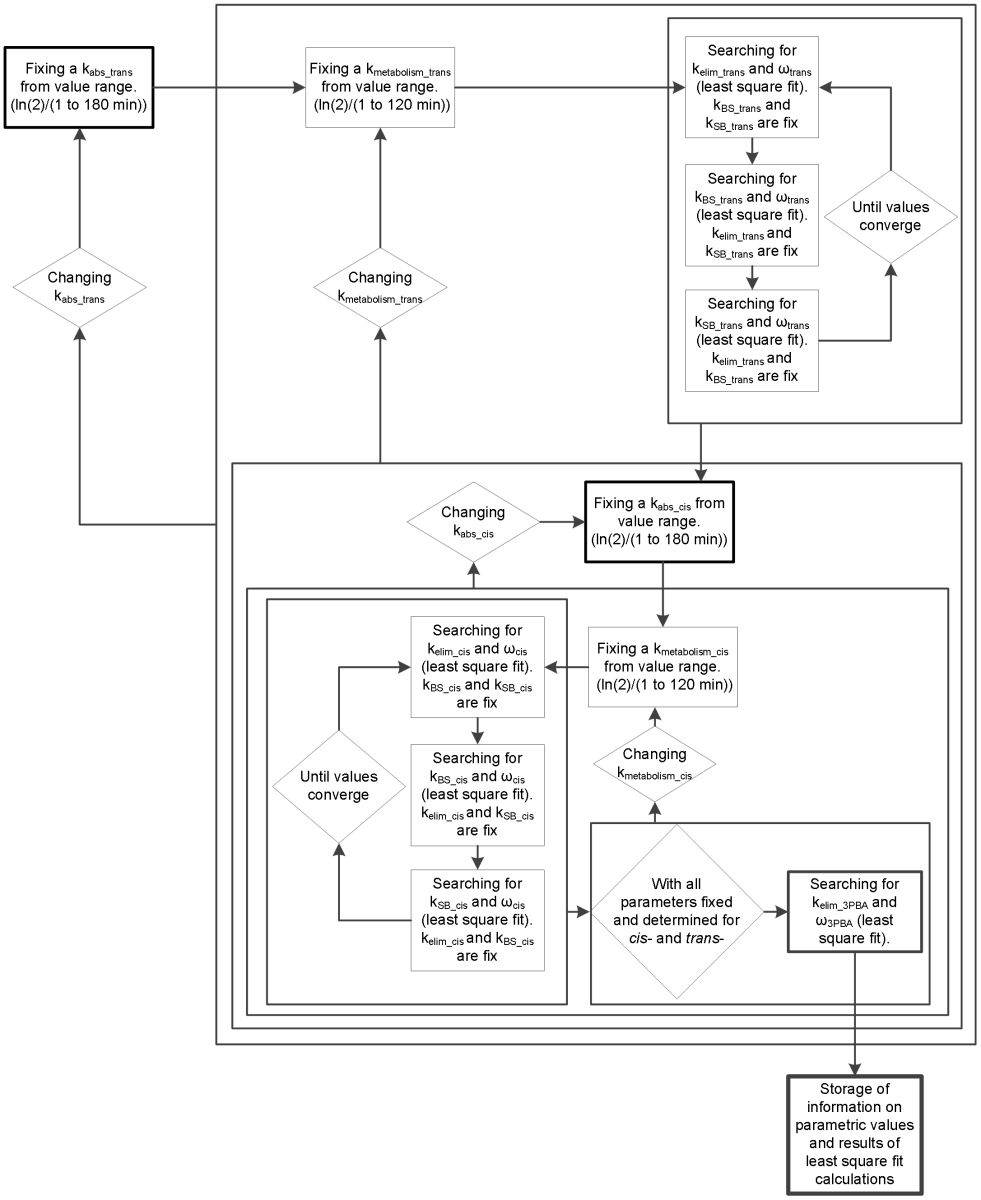
Algorithm. Algorithm for the determination of parameters values of the model with experimental data of urine excretion profile.

A mathematical computer programming has been established to determine model parameter values (Table 2), using Mathcad software (version 14.0.1.286). A specific sequence of determination of parameter values for permethrin and cypermethrin model was programmed with successive iteration, by best-fit adjustments (least square fits) to observed time course data available in the literature [31]. Each step of the determination of model parameter values is summarized in Figure 2.

**Table 2:** Parameter values of the model based on fits to the data of Woollen et al. (1992) on both average and individual urinary excretion time courses of *trans*-DCCA, *cis*-DCCA and 3-PBA following an oral and dermal exposure in volunteers.

Analyte	Parameters		Mean^a^ (range)^b^
***Trans-permethrin***	Transfer rate constants (h^−1^)	k_BS_trans_	10.4 (10.4–20.8)
		k_SB_trans_	0.072 (0.058–0.186)
		k_metabolism_trans_	20.8 (8.32–41.6)
		(k_elim_trans_)	0.157 (0.157–0.650)
		k_abs_oral_trans_	0.457 (0.320–0.671)
		k_abs_dermal_trans_	0.033 (0.037–0.066)
	% of administered molar dose as *trans*-DCCA in urine	% related to ω_trans_	18.4 (13.7–23)
	Dermal absorption fraction	f_abs_dermal_trans_	0.820 (0.63–1.23)
***Cis-permethrin***	Transfer rate constants (h^−1^)	k_BS_cis_	3.20 (0.457–10.4)
		k_SB_cis_	0.041 (0.045–0.141)
		k_metabolism_cis_	13.9 (2.08–6.93)
		(k_elim_cis_)	0.184 (0.277–0.462)
		k_abs_oral_cis_	0.317 (0.310–0.473)
		k_abs_dermal_cis_	0.047 (0.043–0.052)
	% of administered molar dose as *cis*-DCCA in urine	% related to ω_cis_	9.51 (6.35–11.3)
	Dermal absorption fraction	f_abs_dermal_cis_	1.25 (0.85–1.99)
**3-PBA**	Transfer rate constants (h^−1^)	(k_elim_3PBA_)	0.095 (0.095–0.462)
	% of administered molar dose as 3-PBA in urine	% related to ω_3PBA_	12.9 (9.19–18.8)

a The reported mean parameter values are the values giving the best fit (÷^2^) to average experimental time course data of Woollen et al. (1992) (n = 6).

b The range of parameter values reported correspond to the minimum and the maximum parameter values giving the best fit (χ^2^) to average and individual experimental data of Woollen et al. (1992) from 4 volunteers. Individual parameter values for two volunteers were not selected because of the presence of incomplete voids.

#### Determination of the analytical solutions

First, differential equations were derived from the model shown in Figure 1. The differential equations have been solved in order to obtain the analytical solutions representing the urinary excretion rate (M(t)×k_MU_; expressed as QU(t) in equation) and the cumulative urinary excretion (U(t)) of cis-DCCA, trans-DCCA and 3-PBA metabolites (equations 1 to 4 of the Appendix S2). These mathematical equations provide, for a given time t, the urinary excretion rate and cumulative urinary excretion of metabolites following a single exposure to permethrin or cypermethrin. These steps were essential to reduce computation time. The resolution of differential equations results in a total of six equations and 14 parameters to be determined. Some parameters correspond to a combination of individual transfer rate constants in the model (Table 1).

The available experimental data of Woollen et al. [31] were used to find numerical values of parameters. Six urinary excretion profiles are associated with each volunteer orally exposed in the study of Woollen et al. [31]: a urinary excretion rate profile and a cumulative urinary excretion profile for *cis*-DCCA, *trans*-DCCA and 3-PBA metabolites. All these profiles were used in order to find a set of parameter values for each volunteer orally exposed. Then, profiles describing excretion of the same metabolites following a dermal exposure were used to find specific parameter values associated with this route-of-exposure. Parameters describing the kinetics following oral absorption of cypermethrin were derived first in order to set all the parameters describing the internal kinetics because the same parameters were also used to describe the kinetics following dermal exposure, except for the absorption fraction and rate.

#### Sensitivity analysis of parameters

A sensitivity analysis was then performed on each parameter in order to determine time windows of urinary excretion profiles where parameters have influence on simulated curves. These time windows are considered in the computer programming process to best-fit and find a specific parameter value.

An initial set of values determined from best-fits to observed data of Woollen et al. [31] on the urinary excretion profiles of *cis*-DCCA, *trans*-DCCA and 3-PBA metabolites in volunteers served as default values in the examination of the influence of each model parameter. Release of the parent compound from storage tissue compartment and elimination rate constant values (k_SB_ and k_elim_) were estimated by log-linear regression on a specific range of experimental points from urinary excretion rate profiles (average experimental points between 66 and 114 h for k_SB_ and between 6 and 30 h for k_elim_ corresponding to the visual inspection of two apparent elimination phases). The other parameters were independently found for each metabolite, by using a built-in function in Mathcad (“genfit”), which provides a best-fit of a general equation to observed experimental data.

The sensitivity analysis was then performed by varying the parameter values to determine their influence on simulated urinary excretion curves. Metabolism (k_metabolism_) and oral absorption (k_abs_oral_) constants were not associated with a time window as they had only an overall influence on simulated urinary excretion curves. Influence of k_BS_ on simulated cumulative urinary excretion curves was observed over the 12–48 h period, and of k_SB_ and k_elim_ on urinary excretion rate curves between 42–114 h and 6–30 h, respectively.

#### Determination of sets of model parameter values following oral absorption

The computer tool verifies multiple combinations of parameter values (in equations 1–4) that fit the experimental data of Woollen et al. [31] on the urinary excretion profiles of cis-/trans-DCCA and 3-PBA metabolites for each exposed volunteer (urinary excretion rate and cumulative urinary excretion of metabolites). A range of variation was set for each parameter on the basis of physiological constraints. More specifically, since elimination of the parent compound from the body is complete within 3 to 5 days, an upper limit value of 1/8000 minutes^−1^ was set for storage and elimination parameters (applied to k_BS_, k_SB_ and k_elim_), considering a unit value of 1 minute^−1^ for all constants in the model. Secondly, an upper limit value of 1/600 minutes^−1^ for the absorption rate constant was set in accordance with normal intestinal transit time (between 10 and 14 hours) [24]. Finally, the upper bound value of the metabolism constant was set at 1/180 minutes^−1^, on the basis of the rapid metabolism of permethrin and cypermethin reported in the literature [32].

Subsequently, in sequence, the computer program selected and fixed an oral absorption constant value (k_abs_oral_) and a metabolism constant value (k_metabolism_) in the parameter value range previously set for the *trans*- isomer. With these fixed constants, the program automatically determined the storage (k_BS_), release from storage (k_SB_), elimination (k_elim_) and omega (k_BM_×k_MU_) rate constants (see Table 1 for more definitions of symbols). This was done by successive iteration of k_BS_ parameters (omega parameter is calculated at each iteration) between predetermined parameter values intervals until simulated curves reached the best-fit (least square fit) to experimental points over specified time windows set on the basis of prior sensitivity analysis. This operation was then performed sequentially for the determination of k_SB_ and ρ (k_elimination_) parameters. With these newly derived values for k_BS_, k_SB_ and k_elim_ parameters, the program was set to re-run these operations until parameters converge to values providing the best adjustments between simulated and experimental points of both the urinary excretion rate profile and cumulative urinary excretion time course of the *trans*-DCCA metabolite. These operations can significantly reduce computation time while determining the best set of parameter values specific to metabolite *trans*-DCCA, using a fixed constant k_abs_oral_ and k_metabolism_. They thus allow skipping calculations of function solutions with sets of parameter values that would have been eliminated anyway in the final selection process.

Once these sets of values were fixed by best-fits to *trans*-DCCA kinetics, parameters were then calculated in the same manner on the basis of *cis*-DCCA kinetics. Oral absorption k_abs_oral_ and metabolism k_metabolism_ constants were fixed and the computer program then automatically determined the storage (k_BS_), release from storage (k_SB_), elimination (k_elim_) and omega (k_BM_×k_MU_) constants. However, physiological constraints were taken into account at this stage, by imposing oral absorption and metabolism rates of the *trans*- isomer that were faster than those of the *cis*- isomer, on the basis of the literature [32], [37], [38], [39], [40], [41], [42], [43], [44]. In addition, the constant describing release of the parent compound from storage compartment, k_SB,_ was constrained to be slower for the *cis*- isomer because of its more lipophilic property [31], [33], [36], [45].

As a last step in the iterative process, best-fits to the experimental data of Woollen et al. [31] on the urinary excretion rate profile and cumulative urinary excretion time course of the 3-PBA metabolite were programmed to determine the elimination rate (k_elim_ (k_MU_+k_MF_)) and omega constant (ω (k_BM_×k_MU_)) (within a predetermined parameter values interval), common to both the DCCA and 3-PBA kinetics. It was assumed that the same k_metabolism_ constants could be used to describe both the formation of DCCA (*cis*- and *trans*-) and 3-PBA metabolites, considering that intermediate metabolites in the 3-PBA metabolism pathway, such as 3-phenoxybenzoic alcohol (CH_2_OH), are minor metabolites.

The computer program performed these iterations in loop, by changing parameter values of k_abs_oral_ or k_metabolism_ (for *cis*- and *trans*- isomers) and subsequent parameters until all parameter values from predetermined intervals for k_abs_oral_ and k_metabolism_ constants of both isomers had been tested. Once these steps were completed, the computer program stored all these sets of parameter values and associated reliability factors (least square fit) in a matrix.

#### Determination of the best set of model parameter values following oral absorption

The created matrix contained more than a hundred thousand sets of parameter values. Each set of parameter values was associated with six reliability factors, one for each of the six simulated urinary excretion profiles (rate and cumulative excretion profiles of each of the three modeled metabolites). A classification of sets of parameter values (from best to worst) was then performed for each simulated urinary excretion profile and associated reliability factor, such that each set of parameter values was given six ranking values. These six ranking values were summed to derive a new combined ranking, the latter of which provided the retained best set of parameter values for an oral exposure scenario (smallest combined ranking value).

#### Determination of sets of model parameter values following dermal absorption

After setting best-fit parameter values describing the internal kinetics on the basis of prior computations, sets of parameter values specific to the dermal exposure route were derived (i.e., the dermal absorption rate and fraction) in a similar manner as previously described. This was performed by successive iterative best-fits to the time course data of Woollen et al. [31] in each of the volunteers exposed dermally to cypermethrin. In this iterative process, the dermal absorption fraction was first fixed and the dermal absorption constant was left to vary. Subsequently, dermal absorption fraction value was varied to provide a range of sets of parameter values in a matrix and in turn identify the best set of dermal parameter values. These operations were done independently for the cis- and trans- isomers.

### Model simulation and evaluation

Once the parameter values of the model were determined, the system of differential equations representing the complete model of the kinetics of permethrin/cypermethrin and their metabolites was numerically solved in MathCad 14 (PTC (Parametric Technology Corporation), Needham, MA, USA) using the Runge-Kutta method. The ability of the model to reproduce sets of experimental data other than the ones used to derive the parameter values was tested. For this purpose, the various time course data of Tomalik-Scharte et al. [16] in volunteers dermally exposed to permethrin were used.

## Results

### Model parameters and simulation

**Figure 3:**
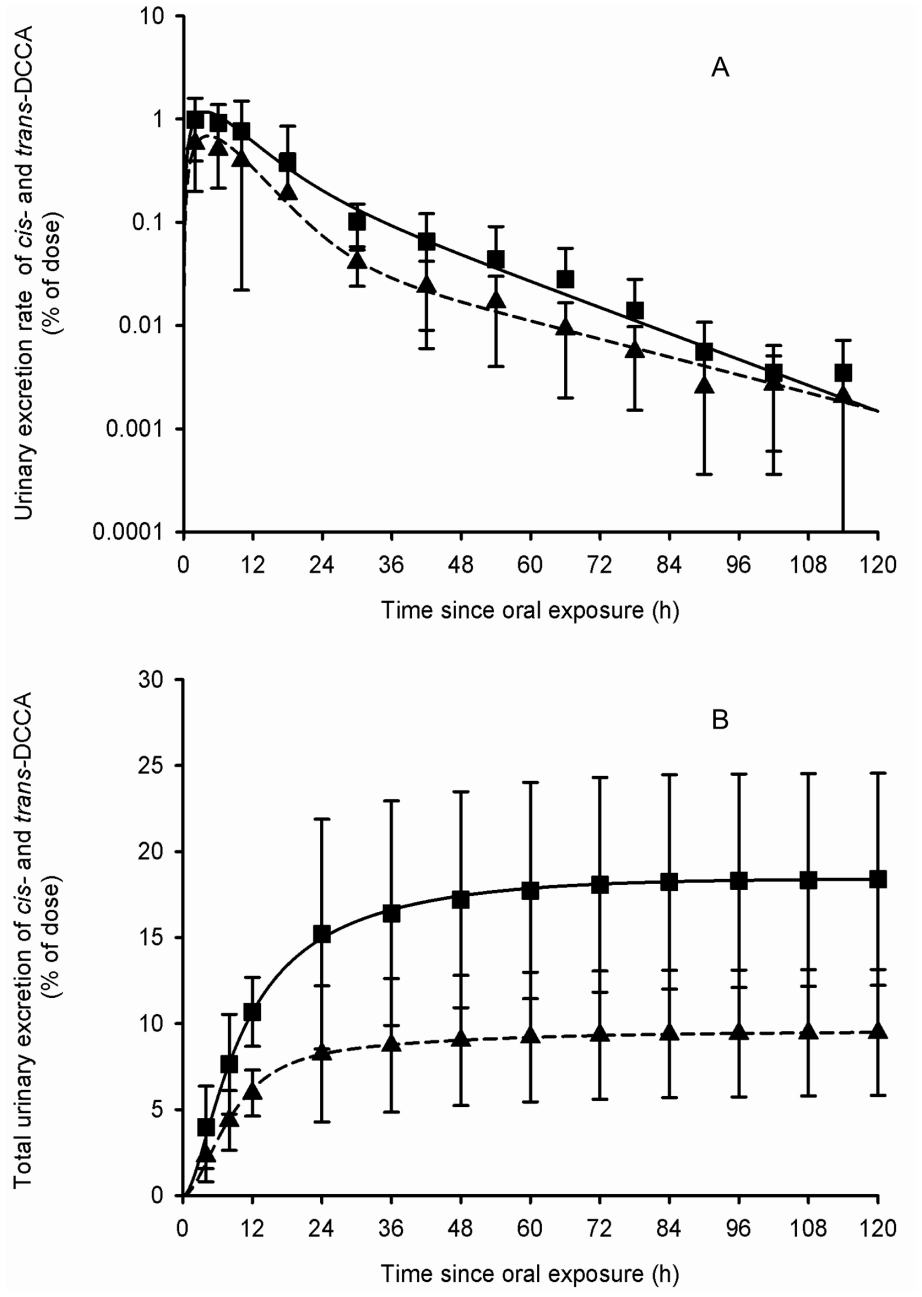
Comparison of model simulations with experimental data for *cis*- and *trans*-DCCA (volunteers orally exposed). Comparison of model simulations (lines) with experimental data of Woollen et al. (1992) (symbols) on the average time courses of *cis*- and *trans-DCCA* excretion rate (A) and cumulative excretion (B) (% of administered dose) in volunteers orally exposed to 3.3 mg of cypermethrin. Triangle and square symbols represent average experimental values for *cis*- and *trans*-DCCA, respectively, and vertical bars the experimental standard deviation (n = 6).

**Figure 4:**
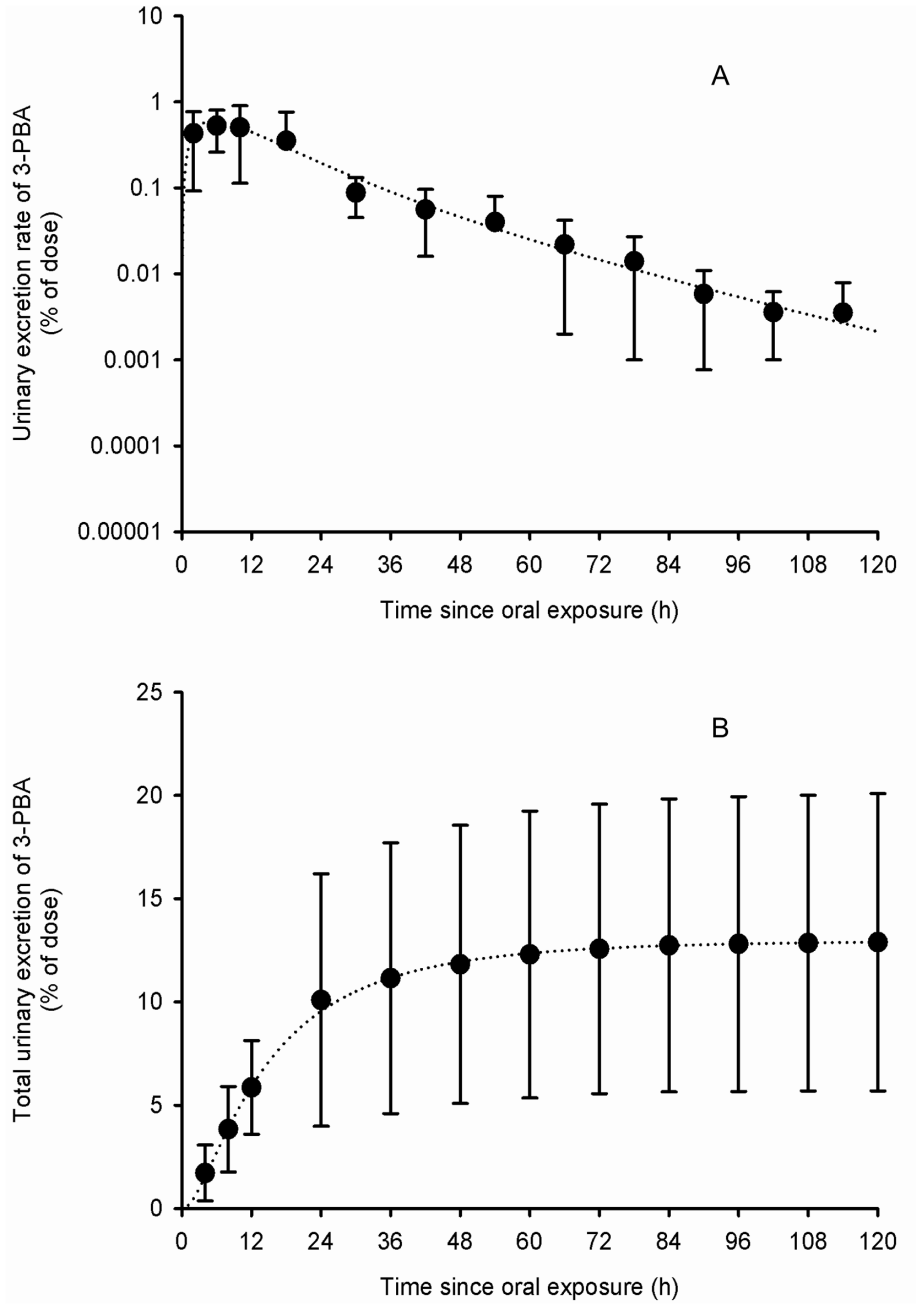
Comparison of model simulations with experimental data for 3-PBA (volunteers orally exposed). Comparison of model simulations (lines) with experimental data of Woollen et al. (1992) (symbols) on the average time courses of 3-PBA excretion rate (A) and cumulative excretion (B) (% of administered dose) in volunteers orally exposed to 3.3 mg of cypermethrin. Symbols represent average experimental values and vertical bars the experimental standard deviation (n = 6).

**Figure 5:**
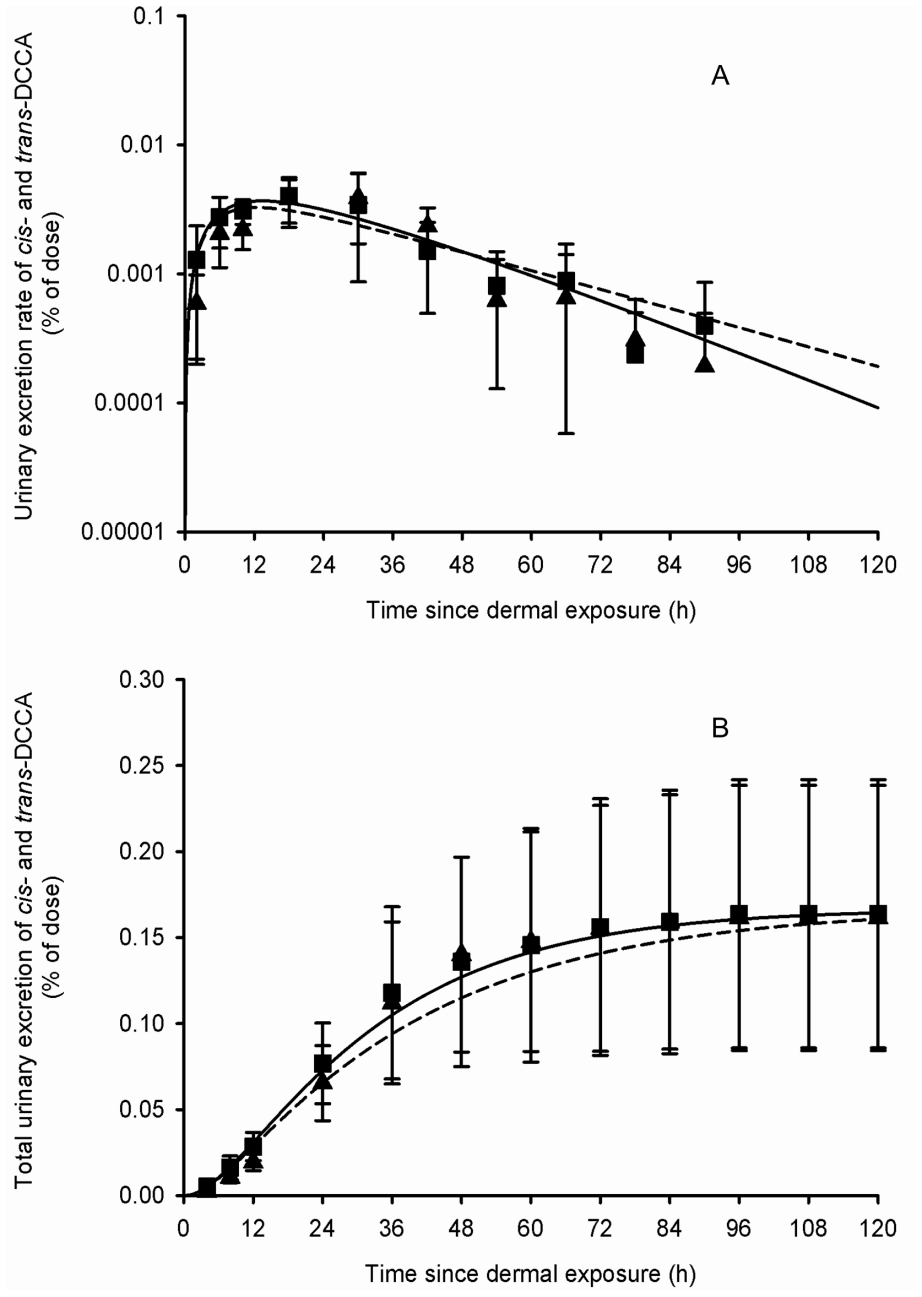
Comparison of model simulations with experimental data for *cis*- and *trans*-DCCA (volunteers dermally exposed). Comparison of model simulations (lines) with experimental data of Woollen et al. (1992) (symbols) on the average time courses of *cis*- and *trans-DCCA* excretion rate (A) and cumulative excretion (B) (% of applied dose) in volunteers dermally exposed to 31 mg of cypermethrin. Triangle and square symbols represent average experimental values for *cis*- and *trans*-DCCA, respectively, and vertical bars the experimental standard deviation (n = 6).

**Figure 6:**
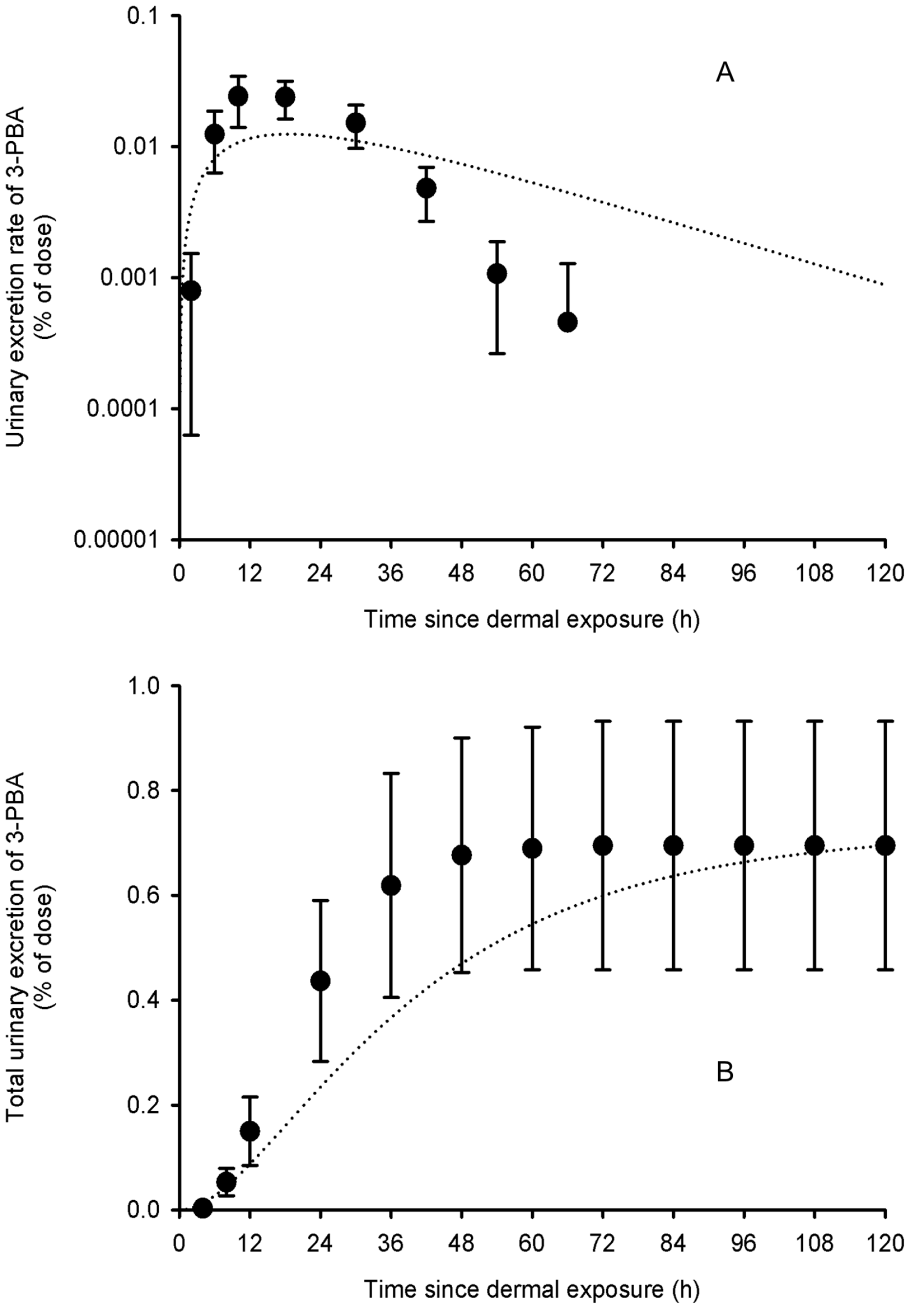
Comparison of model simulations with experimental data for 3-PBA (volunteers dermally exposed). Comparison of model simulations (lines) with experimental data of Woollen et al. (1992) (symbols) on the average time courses of 3-PBA excretion rate (A) and cumulative excretion (B) (% of applied dose) in volunteers dermally exposed to 31 mg of cypermethrin. Symbols represent average experimental values and vertical bars the experimental standard deviation (n = 6).

Table 2 presents the set of parameter values obtained from adjustments to the average and individual data of Woollen et al. [31]. Figures 3–6 show that these sets of parameter values (in equations 1–4 of the Appendix S2) provided very good fits to the experimental data of Woollen et al. [31] on the excretion time courses of *trans*-DCCA, *cis*-DCCA and 3-PBA in volunteers orally and dermally exposed to cypermethrin. However, in the specific case of the dermal exposure, a multiplicative factor had to be applied to represent the appropriate total excretion levels of 3-PBA. The average oral absorption fraction was fixed to 0.80. The average dermal absorption fraction for *cis*- and *trans*-cypermethrin was estimated to be 1.25 and 0.82%, on the basis of model adjustments.

### Model evaluation

**Figure 7:**
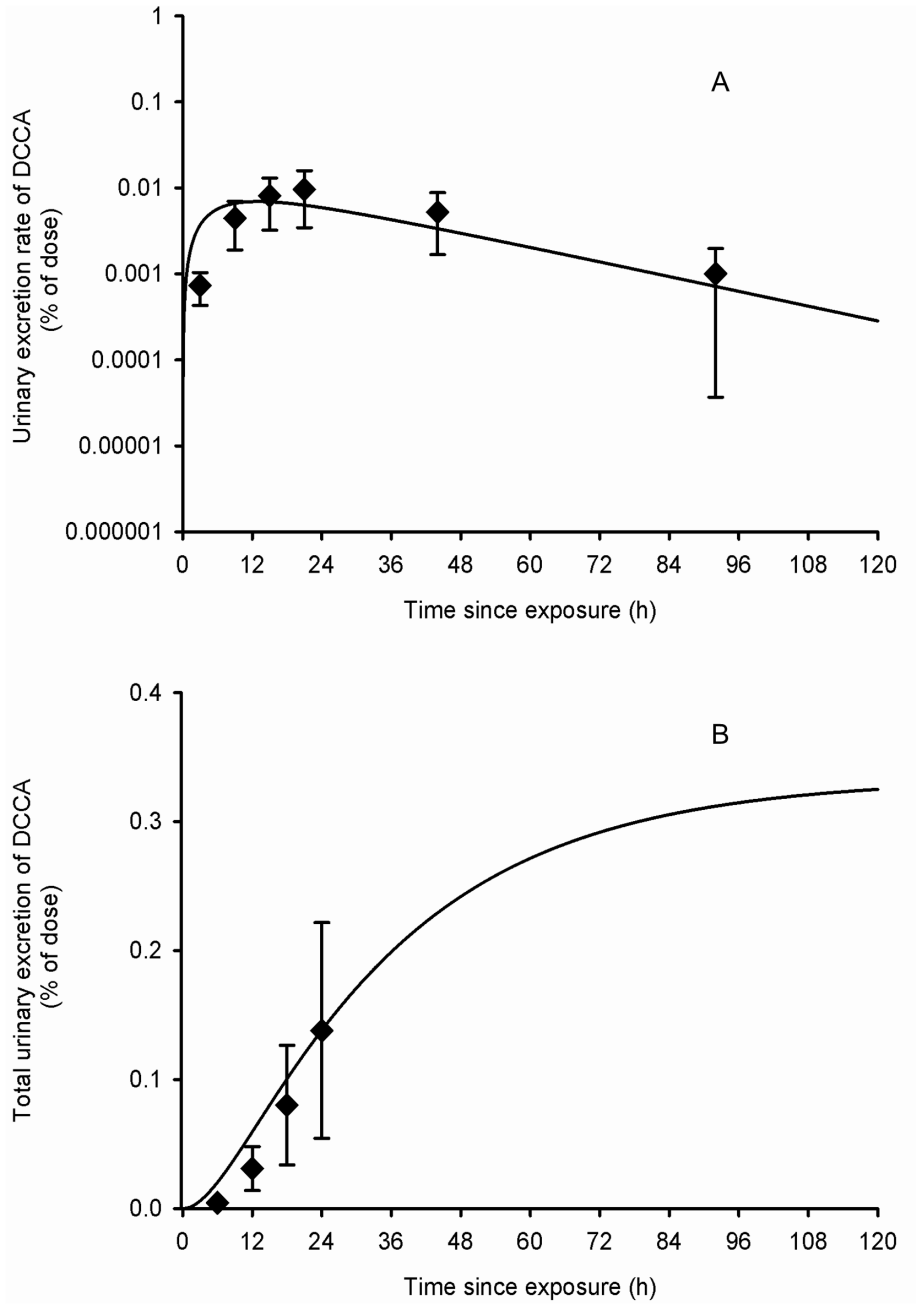
Comparison of model simulations with experimental data for DCCA (volunteers dermally exposed). Comparison of model simulations (lines) with experimental data of Tomalik-Scharte et al. (2005) (symbols) on the average time courses of DCCA excretion rate (A) and cumulative excretion (B) (% of applied dose) in healthy volunteers following a whole-body dermal application of a cream containing 3 g of permethrin. Diamond symbols represent average experimental values and vertical bars the experimental standard deviation (n = 6).

**Figure 8:**
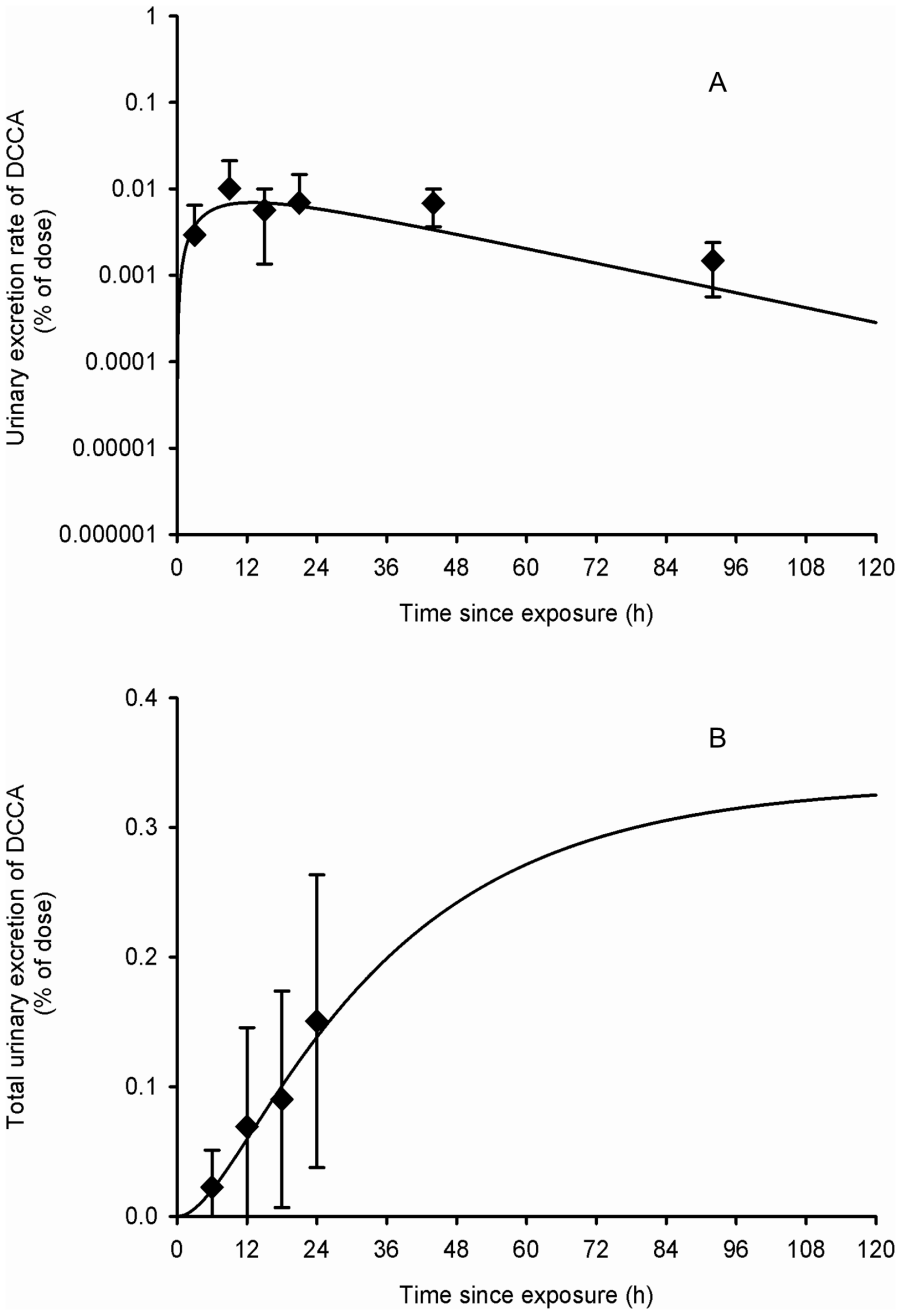
Comparison of model simulations with experimental data for DCCA (scabies patients dermally exposed). Comparison of model simulations (lines) with experimental data of Tomalik-Scharte et al. (2005) (symbols) on the average time courses of DCCA excretion rate (A) and cumulative excretion (B) (% of applied dose) in scabies patients following a whole-body dermal application of a cream containing 3 g of permethrin. Diamond symbols represent average experimental values and vertical bars the experimental standard deviation (n = 6).

**Figure 9:**
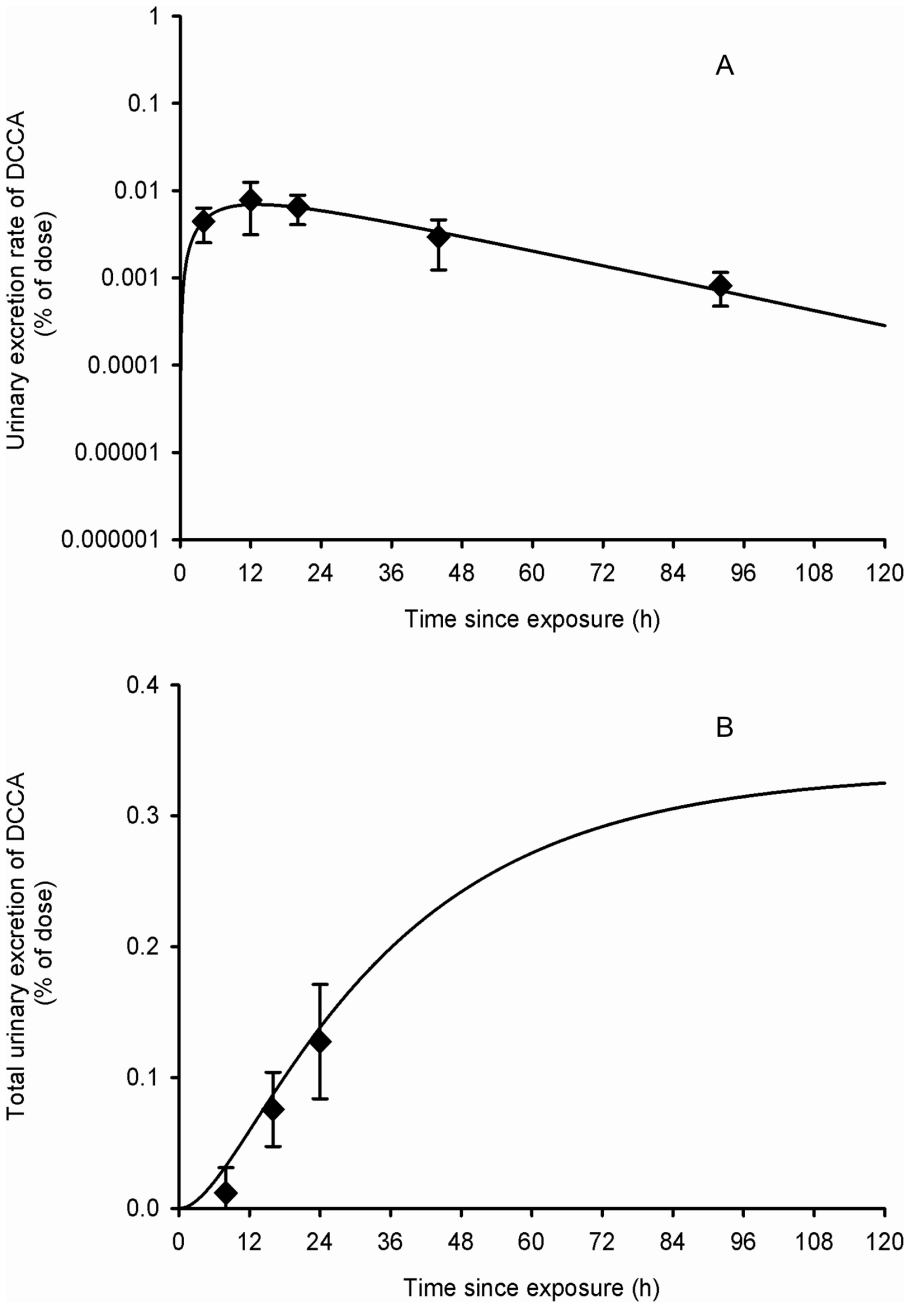
Comparison of model simulations with experimental data for DCCA (volunteers dermally exposed). Comparison of model simulations (lines) with experimental data of Tomalik-Scharte et al. (2005) (symbols) on the average time courses of DCCA excretion rate (A) and cumulative excretion (B) (% of applied dose) in healthy volunteers following a dermal application of 215 mg of a permethrin solution on the scalp. Diamond symbols represent average experimental values and vertical bars the experimental standard deviation (n = 6).

Using parameter values based on the kinetics of cypermethrin and its metabolites (Table 1), the model also provided a very good fit to independent sets of data by Tomalik-Scharte et al. [16] on the urinary excretion time courses of the sum of *trans*- and *cis*-DCCA in individuals dermally exposed to permethrin (Figures 7–9). The model developed from excretion time course data in cypermethrin-exposed volunteers thus appears suitable to simulate the kinetics of common metabolites in permethrin-exposed individuals. Even the dermal absorption rate and fraction of permethrin were kept as determined for cypermethrin.

### Model inferences

The model predicts that, following a single oral exposure as in Woollen et al. [31] (50∶50 *cis*∶*trans*), with an absorption fraction of 0.8 and absorption rate constant of 0.32/0.46 h^−1^ (*cis*/*trans*) (half-life of 131/91 min) as reported in Table 2, the predicted time course of the parent compound in blood B(t) shows maximum level 15/9 min postexposure, which represents 0.69/0.55% of the exposure dose (0.86/0.69% of the absorbed dose). Because of an early partial storage in tissues and a relatively slower return from these tissues to blood, simulated elimination of the parent compound from blood follows a bi-exponential pattern with a more rapid phase followed by a slower phase (Figs. 2–5). Model simulations predict a partial transfer of the parent compound from blood to storage tissues S(t), where the maximum storage level represents 5.8/10.2% of the exposure dose (7.2/12.8% of the absorbed dose). Transfer of the parent compound from storage tissues S(t) to blood B(t) is associated with a predicted half-life for this process of 16.9/9.6 h. The parent compound is also readily and extensively metabolized in the body and its metabolites are rapidly eliminated once formed; the maximum level of total metabolites M(t) is reached 4.3/3.9 h postexposure and represents 6.7/10.1% of the exposure dose (8.3/12.7% of the absorbed dose). The cumulative urinary excretion time courses of metabolites show that the different metabolites are rapidly eliminated; the model predicts that 65.2/57.9 and 86.7/81.3% of total amounts recovered in urine are excreted during the first 12 and 24 h postexposure, respectively. Asymptotically, urinary *cis*-DCCA, *trans*-DCCA and 3-PBA represent respectively 9.5, 18.4 and 12.9% of the exposure dose (11.9, 23.1 and 16.2% of the absorbed dose).

Following a single dermal exposure (50∶50 *cis*∶*trans*), using an absorption fraction of 0.01 and an absorption rate constant of 0.033/0.047 h^−1^ (half-life of 20.9/14.7 h) as reported in Table 2, the predicted time courses of the parent compound in blood B(t) and in storage tissues S(t), and total metabolites in the body M(t) show respective peak levels 0.38/0.23, 30.2/20.98, 12.4/13.5 h postexposure. Since the dermal absorption of permethrin and cypermethrin is small and relatively slow compared to metabolism *cum* elimination, maximum values for B(t), S(t) and M(t) only amount to 0.00096/0.00075, 0.034/0.061 and 0.023/0.044% of the exposure dose, respectively (0.096/0.075, 3.44/6.07 and 2.26/4.40% of the absorbed dose). Also, because dermal absorption of the parent compound is slow compared to its biotransformation, a dynamic equilibrium is quickly reached between the skin compartment D(t) and blood compartment B(t). Consequently, B(t) begins its attrition at the rate of the absorption rate constant of 0.033/0.047 h^−1^ (half-life of 20.9/14.7 h), which is not the case following an oral exposure where absorption is more rapid than elimination processes. From the cumulative urinary excretion time courses of metabolites, the model predicts that 17.1/18.5 and 40.7/43.7% of total amounts recovered in urine are excreted during the first 12 and 24 h, respectively, following a dermal exposure. Asymptotically, urinary *cis*-DCCA, trans-DCCA and 3-PBA represent 0.12, 0.23 and 0.16% of exposure dose (11.9, 23.1 and 16.2% of the absorbed dose; these latter excretions are identical to those after oral exposure, as should be). These simulations show that following a dermal exposure to permethrin or cypermethrin, the absorption rate constant governs the overall urinary excretion rate of the metabolites, because the dermal absorption rate, k_abs_dermal_, is small compared to the biotransformation rate and renal clearance (represented in the model by k_BM_ and k_MU_, respectively). According to model predictions, to recover half of the absorbed dose of permethrin or cypermethin eventually excreted in urine as metabolites takes 31.2 h following a single dermal application compared to 8.6 h following oral exposure.

**Figure 10:**
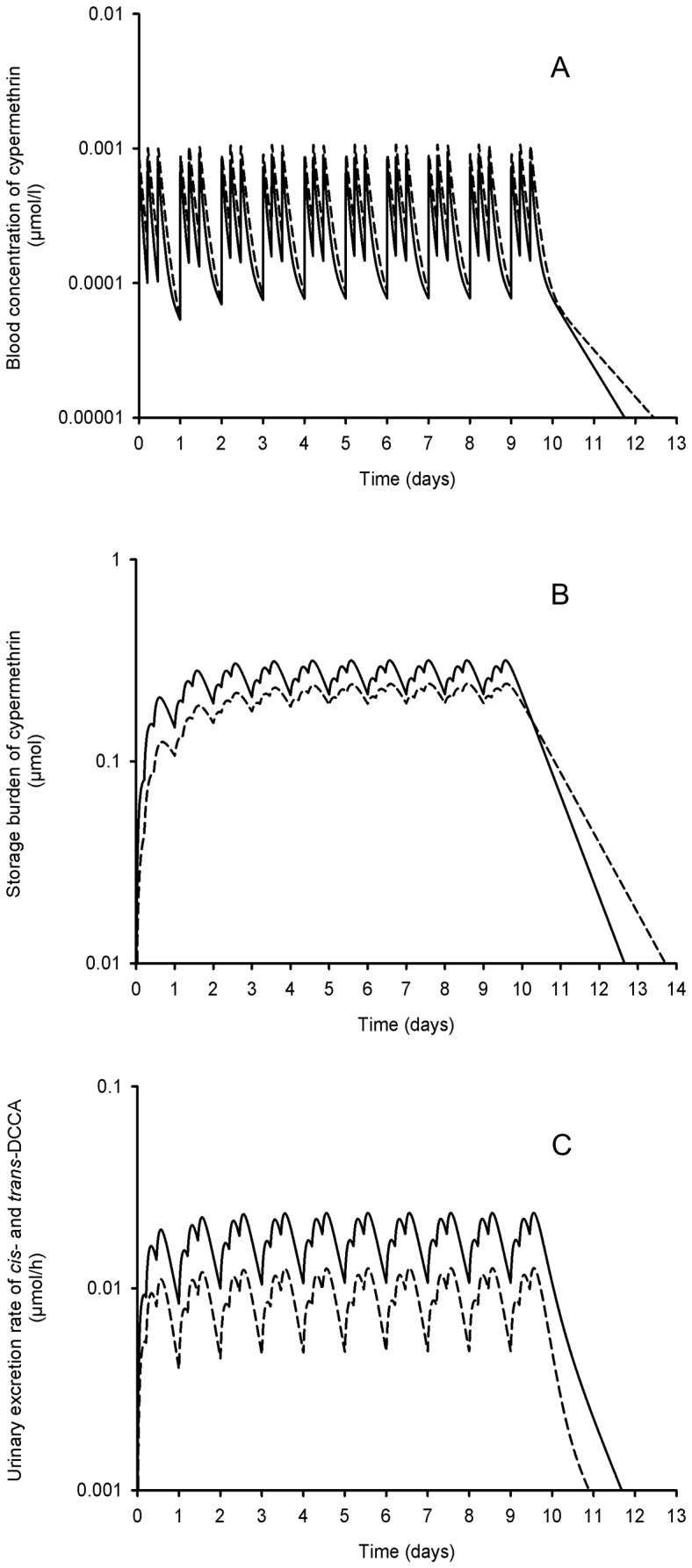
Model simulations of cypermethrin and DCCA in blood, storage tissues and urine following repeated oral exposure. Model simulations (lines) of the time courses of cypermethrin in blood (B(t)) (A) and storage tissues (S(t)) (B) as well as *trans*- and *cis*-DCCA in urine (U(t); solid and dotted lines, respectively) (C) following a repeated oral exposure, 3 times per day (at 7:30 am, 12: 30 am and 6:30 pm), during 10 consecutive days to a dose corresponding to 1/10 of the dose administered by Woollen et al. (1992) (0.33 mg/day).

The proposed model can also predict the time evolution of permethrin or cypermethrin and their metabolites in the body and excreta following repeated exposures. Simulations of a repeated oral exposure, three times per day (7:30 am, 12:30 am and 6:30 pm), over 10 consecutive days (Figure 10) has shown a daily increase in burdens of the parent compound in blood B(t) and in storage tissues S(t) and total body metabolites M(t) as well as metabolites excreted in urine U(t). However, there is little day to day variations in daily minimum and maximum levels because a steady-state equilibrium is rapidly reached.

## Discussion

### Model development

A toxicokinetic model was developed in this study to assess exposure to pyrethroids in the general population. The model describes the time courses of urinary biomarkers of permethrin and cypermethrin under different exposure routes, with a focus on the oral route, the main-of-entry in the general population. The model was built for biological monitoring purposes and not, for instance, to uncover the links between target tissue concentrations and the appearance of health effects. It predicts the essential features of the kinetics of permethrin and cypermethrin and its metabolites in humans without the need for detailed knowledge of internal physiological processes.

The model thus simulates the storage and breakdown of permethrin and cypermethrin to their common *trans*- and *cis*-DCCA metabolites and 3-PBA counterpart, based on metabolism data [46], and ensuing rapid urinary elimination, in line with available human profile data in volunteers [31], [32], [33], [34], [47]. Given the similar observed excretion kinetics of the *trans*- and *cis*-DCCA originating from both permethrin and cypermethrin, a single model, with common parameter values, was used and provided very good predictions to available time course data in the literature [16], [31].

The conceptual model was developed by separating the kinetics of the *cis*- and *trans*- isomers of cypermethrin and permethrin. Total amounts of 3-PBA metabolite originate from the addition of the biotransformation of *cis*- and *trans*- forms of the parent compound. This allows accounting for kinetic differences in the rate of absorption, storage, metabolism and elimination of the *cis*- and *trans*- isomers, as pointed out by some authors [31], [32], [33], [36].

In line with the literature on cypermethrin kinetics, absorption of the parent compound was modeled to be faster for the *trans*-isomer as compared to the *cis*-isomer (according to the JMPR [37], [38]). Storage of the parent compound was also modeled to be more significant for the *cis*-form of the parent compound, in accordance with published data, which indicates that the *cis*- form of permethrin and cypermethrin has a greater affinity for fat tissues than the *trans*-isomer [31], [32], [36]. In particular, in an experimental study in rats, Crawford et al. [32] found that amounts of the *cis*-isomer of cypermethrin was significantly higher in fat tissues then those of the *trans*-isomer following a single oral exposure. The urinary excretion rate time courses of Woollen et al. [31] confirm this phenomenon. Indeed, assuming a fast metabolism rate (half-life of about 30 min to 2 h), the time profiles between 30 h and 114 h post-dosing suggest a second slower elimination phase, resulting from the release of the parent compound from a storage compartment (this release rate being slower than the metabolism rate).

Metabolism of permethrin and cypermethrin was also modeled to be very rapid, again in line with the literature [32], [48]. It seems that the main process of metabolism of the *trans*-isomer is hydrolysis [32], [40]. Hydrolysis is much more effective for the *trans*-isomer than the *cis*-isomer of permethrin and cypermethrin, which appears to be more stable in the body [32], [40]. The *cis*-isomer, less hydrolyzed, has been documented to be partly metabolized by oxidation [32].

Furthermore, according to Casida et al. [41], the *trans*-form is hydrolyzed 50 times faster than the *cis*-isomer. Ross et al. [42] mentioned that carboxylesterase hCE-1 and hCE-2 respectively hydrolyze 8 and 28 time more rapidly the *trans*-permethrin than the *cis*-isomer, while Nishi et al. [43] reported that they are respectively 12 and 5 times more efficient in metabolizing the *trans*-isomer of permethrin form and 14 and 29 times more effective in metabolizing the *trans*-isomer of cypermethrin.

A different metabolism process of the *cis*-isomer compared to the *trans*-isomer was also associated with a greater fecal elimination of the *cis*-form; our model accounts for this feature. Crawford et al. [32] showed that more *cis*- isomer is found in feces after an oral exposure in rats compared to the *trans*- isomer. Furthermore, Hutson et al. [39] stated that oxidation at peripheral sites, while leaving the ester bond intact, facilitates phase II conjugation reactions, leading to biliary and fecal elimination of the esters. The urinary excretion profiles of *trans*-DCCA and *cis*-DCCA are relatively similar, but it should be noted there is an about 2-fold ratio between the cumulative urinary excretion of *trans*-DCCA and *cis*-DCCA after an oral exposure (for a *cis*/*trans* ratio of 50∶50 for the parent compound in Woollen et al. [31]).

Moreover, according to Kaneko et al. [35], other metabolites than the monitored *trans*-DCCA, *cis*-DCCA and 3-PBA may be formed. Thus, one mol of 50∶50 (*cis*∶*trans*) permethrin or cypermethrin may not create ½ mol of *cis*-DCCA, ½ mol of *trans*-DCCA and 1 mol of 3-PBA and its phenoxy precursors (4-OH-3PBA), justifying the added compartment for unobserved metabolites in the model (Figure 1).

It should be noted that the urinary excretion time courses observed by Woollen et al. [31] after a dermal exposure slightly differs from the observed profiles after an oral exposure. Indeed, the dermal absorption rate is slower than the oral absorption rate. However, a ratio of *trans*- to *cis*-DCCA cumulative excretion equal to unity was observed following a dermal exposure as compared to a ratio value of 2 after an oral exposure. This could be related to a different metabolism process according to the route of exposure [31].

### Determination of parameter values

One of the key aspects of the modeling process was that the mathematical program developed to determine parameters allowed to establish ranges of values using an iterative process, with upper and lower ranges being based on physiological constraints. Due to lack of kinetic data on pyrethroids in the human body and inter-individual variations, it was necessary to determine such model parameters by a computer process. The experimental data of Woollen et al. [31] were found to provide most comprehensive human time courses to determine these parameter values. With the 14 different parameters in the model to represent the excretion kinetics of *trans*-DCCA, *cis*-DCCA and 3-PBA, about 10^23^ sets of parameter values were possible (number based on the prior constraint that each of the determined rate values fell within the set physiological and mathematical limits and considering that the unit value of the model is 1 min). The mathematical program developed ensured a determination of these various possibilities within a reasonable time frame and fixed the best set of parameter values.

We used a deterministic method of global parameter optimization to determine the best set of parameter values. The computer program has been designed to find the best least square fit to the experimental time course data and ensuing parameter values. However, a shortcut was set up in the codes of the computer program that reduces the computation time for determining the best set of parameter values. This shortcut was introduced through sensitivity analysis, which identifies time windows on the course curves where parameters have an influence on the simulated values. To ensure the determination of the best set of parameters, an initial value must be given to the k_elim_ and k_sb_ constants, the only constants that can be immediately derived from the course curves. These initial values were estimated by log-linear regression on a specific range of experimental time-points (a necessary step to set an initial best-fit). However, preliminary tests with other initial values have shown that the retained programming procedure was the one providing the best-fits to the available experimental data.

It was found that release of the parent compound from storage tissues *k_SB_* was slower than the metabolism rate *k_metabolism_*, indicating that *k_SB_* value governed the slope of the excretion rate time courses of the metabolites at later time points post-exposure. Furthermore, not only the metabolism rate, but also the storage rate of the parent k_BS_ was quite rapid. According to the conceptual model, when the product reaches blood, it can be metabolized or stored. A fast storage is required once the parent compound reaches blood; otherwise a slow storage rate combined with a fast metabolism rate would have resulted in no storage of the compound, which would be contrary to the available experimental data showing the accumulation of the parent compound in adipose tissues [32].

It was also found that following oral absorption, the absorption rates of the *cis*- and *trans*-isomers of permethrin and cypermethrin (k_abs_oral_cis_ and k_abs_oral_trans_) were not the limiting-step in the urinary excretion kinetics of the monitored *trans*-DCCA, *cis*-DCCA and 3-PBA metabolites, since the range of variation of this parameter values was based on the rapid intestinal transit time (<3 h). On the other hand, the slower dermal absorption (k_abs_derrmal_cis_ and k_abs_derrmal_trans_) had an influence on the output rate of the metabolites in urine and hence the shape of the excretion time course curves.

### Model simulations and inferences

The model can be used to predict the time courses of permethrin and cypermethrin and their metabolites under different exposure scenarios (oral, dermal and/or inhalation, single or repeated intermittent or continuous exposures). The model provided very good fits the all the oral time course data available and, following dermal exposure, a good fit was observed to the *trans*- and *cis*-DCCA time courses (or total DCCA) [16], [31]. Hence, parameters common to the description of both the *trans*- and *cis*-DCCA time courses also provided good fits to the 3-PBA counterpart. However, fits to the 3-PBA time courses of Woollen et al. [31] following dermal exposure were less consistent with experimental values. This could be explained by differences in metabolism between the oral and dermal route, with a possible significant contribution of the dermis to the metabolism of these compounds [31]. A multiplicative factor had to be applied to represent the appropriate excretion levels of 3-PBA with time following dermal exposure.

Other published data on the time course of biomarkers of exposure to the studied pyrethroids were modeled (not shown), including the data of Kühn et al. [49] on the time courses of cypermethrin metabolites in two exterminator workers during a weekend following an occupational exposure to cypermethrin (*cis*∶*trans* 50∶50). Although a good fit to the data was observed, the exposure dose and route in those workers could not be confirmed. We further modeled the case-study data of Gotoh et al. [36] on the blood concentration-time profile of permethrin over a 9-day period in a subject following admission in a hospital for an oral intoxication (ingestion of 600 mL of an emulsion containing 20% permethrin (*cis*∶*trans* 43.5∶56.5) (not shown in results). However, this patient was suffering from a chronic renal dysfunction and probable renal failure (confirmed by extensively elevated creatinine levels), which probably altered the kinetics compared to observed profiles in volunteers exposed to lower doses.

Further research on the time courses of permethrin and cypermethin and their metabolites in the human body (such as blood) as well as more data on the relative importance of dermal metabolism compared to blood or liver metabolism would help refine or further evaluate the model and variability in parameter values. Cytochrome P450 activity in the skin may contribute to a different metabolism of permethrin and cypermethrin compared to the highly active liver carboxylesterase metabolism (hCE-1 and hCE-2) [41], [42], [43], [44]. Nevertheless, the compartments used in the model such storage tissues and isomer specific compartments (to describe the kinetics of the *trans*- and *cis*-form of permethrin/cypermethrin and their metabolites) were needed to provide adequate simulations of available urinary profiles of the biomarkers of exposure of interest.

Overall, this human data-based model can be used to infer on the time course of *trans*-, *cis*- and 3-PBA biomarkers of exposure under various exposure scenarios, including those likely to occur in the general population chronically exposed mainly through ingestion of residues on certain foods, such as fruits and vegetables. Daily exposure doses (expressed as permethrin or cypermethrin equivalents) can then be predicted in this population from biomonitoring results as in similar studies [27], [50].

## Supporting Information

Appendix S1 **Differential equations used to represent the kinetic model of** ***cis*** **- and** ***trans*** **-permethrin and cypermethrin and their** ***trans*** **-DCCA,** ***cis*** **-DCCA and 3-PBA metabolites.**
(DOCX)

Appendix S2 **Analytical solutions of the differential equations used to represent the urinary excretion rates and the cumulative urinary excretion of** ***trans*** **-DCCA,** ***cis*** **-DCCA and 3-PBA metabolites.**
(DOCX)
