# Supplementary material for: A Novel Toxicokinetic Modeling of Cypermethrin and Permethrin and Their Metabolites in Humans for Dose Reconstruction from Biomarker Data
Source: PLoS One. 2014 Feb 26;9(2):e88517. doi: 10.1371/journal.pone.0088517 (PMC3935837; doi:10.1371/journal.pone.0088517)
Supplement: Appendix S1 — Differential equations used to represent the kinetic model of cis - and trans -permethrin and cypermethrin and their trans -DCCA, cis -DCCA and 3-PBA metabolites. (DOCX) [file pone.0088517.s001.docx]

**Appendix S1**. Differential equations used to represent the kinetic model of *cis*- and *trans*-permethrin and cypermethrin and their *trans*-DCCA, *cis*-DCCA and 3-PBA metabolites.

Symbols describing parameters are defined in Table 1. The same equations apply to both the *cis*- and *trans*-DCCA metabolites.

$\frac{\text{dRT}_{\text{i}}\text{(t)}}{\text{dt}}=-\text{k}_{\text{abs\_inh}}\cdot\text{RT}_{\text{i}}\text{(t)}+\text{g}_{\text{inh}}\text{(t)}$;

$\frac{\text{dD}_{\text{i}}\text{(t)}}{\text{dt}}=-\text{k}_{\text{abs\_dermal}}\cdot\text{D}_{\text{i}}\text{(t)}+\text{g}_{\text{dermal}}\text{(t)}$;

$\frac{\text{dGI}_{\text{i}}\text{(t)}}{\text{dt}}=-\text{k}_{\text{abs\_oral}}\cdot\text{GI}_{\text{i}}\text{(t)}+\text{g}_{\text{oral}}\text{(t)}$;

$\frac{\text{dB}_{\text{i}}\text{(t)}}{\text{dt}}=\text{k}_{\text{abs\_inh}}\cdot\text{RT}_{\text{i}}\text{(t)}+\text{k}_{\text{abs\_dermal}}\cdot\text{D(t)}_{\text{i}}+\text{k}_{\text{abs\_oral}}\cdot\text{GI}_{\text{i}}\text{(t)}-\text{(k}_{\text{BS}}+\text{k}_{\text{BM\_not observed}}+\text{k}_{\text{BM}}\text{)B}_{\text{i}}\text{(t)}+\text{k}_{\text{SB}}\cdot\text{S}_{\text{i}}\text{(t)}$;

$\frac{\text{dS}_{\text{i}}\text{(t)}}{\text{dt}}=\text{k}_{\text{BS}}\cdot\text{B}_{\text{i}}\text{(t)}-\text{k}_{\text{SB}}\cdot\text{S}_{\text{i}}\text{(t)}$;

$\frac{\text{dS}_{\text{i}}\text{(t)}}{\text{dt}}=\text{k}_{\text{BS}}\cdot\text{B}_{\text{i}}\text{(t)}-\text{k}_{\text{SB}}\cdot\text{S}_{\text{i}}\text{(t)}$;

$\frac{\text{dM}_{\text{not observed}}\text{(t)}}{\text{dt}}=\text{k}_{\text{BM\_not observed}}\cdot\text{B}_{\text{i}}\text{(t)}$;

$\frac{\text{dM}_{\text{j}}\text{(t)}}{\text{dt}}=\text{k}_{\text{BM}}\cdot\text{B}_{\text{i}}\text{(t)}-\text{(k}_{\text{MU}}+k_{MF}\text{)M}_{\text{j}}\text{(t)}$;

$\frac{\text{dU}_{\text{j}}\text{(t)}}{\text{dt}}=\text{k}_{\text{MU}}\cdot\text{M}_{\text{j}}\text{(t)}$;

$\frac{\text{dF}_{\text{j}}\text{(t)}}{\text{dt}}=\text{k}_{\text{MF}}\cdot\text{M}_{\text{j}}\text{(t)}$;

where *i* is either *trans*- or *cis*-permethrin or cypermethrin and where *j* is either *trans*-DCCA, *cis*-DCCA or 3-PBA.
