# Supplementary material for: A Novel Toxicokinetic Modeling of Cypermethrin and Permethrin and Their Metabolites in Humans for Dose Reconstruction from Biomarker Data
Source: PLoS One. 2014 Feb 26;9(2):e88517. doi: 10.1371/journal.pone.0088517 (PMC3935837; doi:10.1371/journal.pone.0088517)
Supplement: Appendix S2 — Analytical solutions of the differential equations used to represent the urinary excretion rates and the cumulative urinary excretion of trans -DCCA, cis -DCCA and 3-PBA metabolites. (DOCX) [file pone.0088517.s002.docx]

**Appendix S2**. Analytical solutions of the differential equations used to represent the urinary excretion rates and the cumulative urinary excretion of *trans*-DCCA, *cis*-DCCA and 3-PBA metabolites.

Symbols describing parameters are defined in Table 1. The same QU_DCCA_ and U_DCCA_ analytical solutions apply to both the *cis*- and *trans*-DCCA metabolites:

; **(1)**


 **(2)**

$\begin{matrix} \text{QU}_{\text{3PBA}}=\text{ω}_{\text{3PBA}}\left(\text{η}_{\text{cis}}\text{e}^{\text{λ}_{\text{1cis}}\text{t}}+\text{ε}_{\text{cis}}\text{e}^{\text{λ}_{\text{2cis}}\text{t}}+\text{δ}_{\text{cis}}\text{e}^{-\text{k}_{\text{abs\_oral\_cis}}\text{t}}+\text{K}_{\text{cis}}\text{e}^{-\text{k}_{\text{elim\_3PBA}}\text{t}}\right) \\ +\text{ω}_{\text{3PBA}}\left(\text{η}_{\text{trans}}\text{e}^{\text{λ}_{\text{1trans}}\text{t}}+\text{ε}_{\text{trans}}\text{e}^{\text{λ}_{\text{2trans}}\text{t}}+\text{δ}_{\text{trans}}\text{e}^{-\text{k}_{\text{abs\_oral\_trans}}\text{t}}+\text{K}_{\text{trans}}\text{e}^{-\text{k}_{\text{elim\_3PBA}}\text{t}}\right) \end{matrix}$ **(3)**

$\begin{matrix} \text{U}_{\text{3PBA}}=\text{ω}_{\text{3PBA}}\left(\frac{\text{η}_{\text{cis}}}{\text{λ}_{\text{1cis}}}\text{e}^{\text{λ}_{\text{1cis}}\text{t}}+\frac{\text{ε}_{\text{cis}}}{\text{λ}_{\text{2cis}}}\text{e}^{\text{λ}_{\text{2cis}}\text{t}}-\frac{\text{δ}_{\text{cis}}}{\text{k}_{\text{abs\_oral\_cis}}}\text{e}^{-\text{k}_{\text{abs\_oral\_cis}}\text{t}}-\frac{\text{K}_{\text{cis}}}{\text{k}_{\text{elim\_3PBA}}}\text{e}^{-\text{k}_{\text{elim\_3PBA}}\text{t}}+\text{C}_{\text{cis}}\right) \\ +\text{ω}_{\text{3PBA}}\left(\frac{\text{η}_{\text{trans}}}{\text{λ}_{\text{1trans}}}\text{e}^{\text{λ}_{\text{1trans}}\text{t}}+\frac{\text{ε}_{\text{trans}}}{\text{λ}_{\text{2trans}}}\text{e}^{\text{λ}_{\text{2trans}}\text{t}}-\frac{\text{δ}_{\text{trans}}}{\text{k}_{\text{abs\_oral\_trans}}}\text{e}^{-\text{k}_{\text{abs\_oral\_trans}}\text{t}}-\frac{\text{K}_{\text{trans}}}{\text{k}_{\text{elim\_3PBA}}}\text{e}^{-\text{k}_{\text{elim\_3PBA}}\text{t}}+\text{C}_{\text{trans}}\right) \end{matrix}$ **(4)**

where the following specific η, ε, δ, λ, Κ, and C equations can be applied to either *cis*-DCCA and *trans*-DCCA and ω, k_elim_ equations apply to all three metabolites (thus including 3-PBA):

$\text{η}=\frac{\text{k}_{\text{abs\_oral}}\cdot\text{D}_{\text{abs\_oral}}\left(\text{k}_{\text{SB}}+\text{λ}_{1}\right)}{\sqrt{\text{(k}_{\text{BS}}+\text{k}_{\text{SB}}+\text{k}_{\text{metabolism}}\text{)}^{2}-\text{4k}_{\text{SB}}\text{k}_{\text{metabolism}}}\cdot\left(\text{λ}_{1}+\text{k}_{\text{abs\_oral}}\right)\cdot\left(\text{k}_{\text{elim}}+\text{λ}_{1}\right)}$;

$\text{ε}=-\frac{\text{k}_{\text{abs\_oral}}\cdot\text{D}_{\text{abs\_oral}}\left(\text{k}_{\text{SB}}+\text{λ}_{2}\right)}{\sqrt{\text{(k}_{\text{BS}}+\text{k}_{\text{SB}}+\text{k}_{\text{metabolism}}\text{)}^{2}-\text{4k}_{\text{SB}}\text{k}_{\text{metabolism}}}\cdot\left(\text{λ}_{2}+\text{k}_{\text{abs\_oral}}\right)\cdot\left(\text{k}_{\text{elim}}+\text{λ}_{2}\right)}$;

$\text{δ}=-\left(\frac{\text{η}\cdot\left(\text{λ}_{1}+\text{k}_{\text{elim}}\right)}{\left(\text{k}_{\text{elim}}-\text{k}_{\text{abs\_oral}}\right)}+\frac{\text{ε}\cdot\left(\text{λ}_{2}+\text{k}_{\text{elim}}\right)}{\left(\text{k}_{\text{elim}}-\text{k}_{\text{abs\_oral}}\right)}\right)$;

$\text{K}=\frac{\text{k}_{\text{abs\_oral}}\cdot\text{D}_{\text{abs\_oral}}}{\sqrt{\text{(k}_{\text{BS}}+\text{k}_{\text{SB}}+\text{k}_{\text{metabolism}}\text{)}^{2}-\text{4k}_{\text{SB}}\text{k}_{\text{metabolism}}}\text{(k}_{\text{elim}}-\text{k}_{\text{abs\_oral}}\text{)}}\left(\frac{\left(\text{k}_{\text{SB}}+\text{λ}_{1}\right)}{\left(\text{k}_{\text{elim}}+\text{λ}_{1}\right)}-\frac{\left(\text{k}_{\text{SB}}+\text{λ}_{2}\right)}{\left(\text{k}_{\text{elim}}+\text{λ}_{2}\right)}\right)$;

$\text{λ}_{1}=\frac{1}{2}\left(-\text{(k}_{\text{BS}}+\text{k}_{\text{SB}}+\text{k}_{\text{metabolism}}\text{)}+\sqrt{\text{(k}_{\text{BS}}+\text{k}_{\text{SB}}+\text{k}_{\text{metabolism}}\text{)}^{2}-\text{4k}_{\text{SB}}\text{k}_{\text{metabolism}}}\right)$;

$\text{λ}_{2}=\frac{1}{2}\left(-\text{(k}_{\text{BS}}+\text{k}_{\text{SB}}+\text{k}_{\text{metabolism}}\text{)}-\sqrt{\text{(k}_{\text{BS}}+\text{k}_{\text{SB}}+\text{k}_{\text{metabolism}}\text{)}^{2}-\text{4k}_{\text{SB}}\text{k}_{\text{metabolism}}}\right)$;

$\text{ω}=\text{k}_{\text{BM}}\cdot\text{k}_{\text{MU}}$;

$\text{ρ}=\text{k}_{\text{MU}}+\text{k}_{\text{MF}}$;

$\text{k}_{\text{metabolism}}=\text{k}_{\text{BM}}+\text{k}_{\text{BM\_inconnu}}$;

$\text{C}=-\frac{\text{η}}{\text{λ}_{1}}-\frac{\text{ε}}{\text{λ}_{2}}+\frac{\text{δ}}{\text{k}_{\text{abs\_oral}}}+\frac{\text{K}}{\text{k}_{\text{elim}}}$.
